# Single-Cell RNA sequencing investigation of female-male differences under PAD conditions

**DOI:** 10.3389/fcvm.2023.1251141

**Published:** 2023-09-07

**Authors:** Gloriani Sánchez Marrero, Nicolas Villa-Roel, Feifei Li, Christian Park, Dong-Won Kang, Katherine E. Hekman, Hanjoong Jo, Luke P. Brewster

**Affiliations:** ^1^ Wallace H. Coulter Department of Biomedical Engineering, Georgia Institute of Technology and Emory University, Atlanta, GA, United States; ^2^ Department of Surgery, Emory University School of Medicine, Atlanta, GA, United States; ^3^ Surgical and Research Services, Atlanta Veteran Affairs Medical Center, Decatur, GA, United States

**Keywords:** single-cell RNA sequencing, peripheral arterial disease conditions, sex-specific differences, human aortic endothelial cells, shear stress, substrate stiffness

## Abstract

Peripheral arterial disease (PAD) is an age-related medical condition affecting mostly muscular arteries of the limb. It is the 3^rd^ leading cause of atherosclerotic morbidity. The mechanical environment of endothelial cells (ECs) in PAD is characterized by disturbed blood flow (d-flow) and stiff extracellular matrices. In PAD, the stiffness of arteries is due to decreased elastin function and increased collagen content. These flow and stiffness parameters are largely missing from current models of PAD. It has been previously proven that ECs exposed to d-flow or stiff substrates lead to proatherogenic pathways, but the effect of both, d-flow and stiffness, on EC phenotype has not been fully investigated. In this study, we sought to explore the effect of sex on proatherogenic pathways that could result from exposing endothelial cells to a d-flow and stiff environment. We utilized the scRNA-seq tool to analyze the gene expression of ECs exposed to the different mechanical conditions both *in vitro* and *in vivo*. We found that male ECs exposed to different mechanical stimuli presented higher expression of genes related to fibrosis and d-flow *in vitro*. We validated our findings *in vivo* by exposing murine carotid arteries to d-flow via partial carotid artery ligation. Since women have delayed onset of arterial stiffening and subsequent PAD, this work may provide a framework for some of the pathways in which biological sex interacts with sex-based differences in PAD.

## 1. Introduction

Peripheral arterial disease (PAD) manifests as obstructions in blood flow into the lower limbs. PAD most commonly affects the older populations, with an incidence of 12%–20% in those older than 65 years (1–3). The onset of arterial stiffness and endothelial cell (EC) dysfunction typically begins in the 4^th^ decade (30–40 s), but is delayed approximately 10 years in females compared to males (4). This delay in stiffening corresponds to the subsequent delayed onset of PAD in females (5, 6). PAD is characterized by atherosclerotic lesions in regions commonly affected by disturbed flow (d-flow). D-flow is a combination of low and oscillatory shear stress (OS) at the vessel wall. Furthermore, atherosclerotic lesions have been found in locations where arteries have become stiffer, like those of aged populations (7, 8). Previous work from our group found that d-flow induces a stiffening arteriopathy closely mimicking that of aged arteries (9). The endothelial cells lining PAD arteries can sense both the d-flow of the blood and the stiffness in the vasculature's matrix (10–12). These environmental signals induce EC activation of a proatherogenic pathway often resulting in increased EC permeability, inflammation (EndIT), endothelial-to-mesenchymal transition (EndMT), arterial stiffening, and thrombosis (11). Our research group and others have identified in ECs an important role for fibro-inflammatory pathways in PAD pathology related to thrombospondin-1 (*THBS1*) and cellular communication network factor 2 (*CCN2*), previously better known as connective tissue growth factor (9, 13).

Several comprehensive *in vitro* and *in vivo* studies have been done to understand the molecular pathways that lead to atherosclerosis (9, 14, 15). These mainly involved the transcriptomic bulk analysis of ECs. Recently, single-cell RNA sequencing (scRNA-seq) has become a popular transcriptomics tool because it allows for RNA analysis at a single-cell resolution for a large number of cells. This enables the study of EC heterogeneity as well as the ability to differentiate between cell types included in the sample. Studies using scRNA-seq methods to investigate EC heterogeneity under PAD or atherosclerotic conditions are scarce. One study reported proatherosclerotic EC reprogramming in carotid arteries of wild type mice exposed to d-flow (11), another reported EndMT progression in ECs cultured in stiff matrices (16). Since the characteristic mechanical conditions of PAD involve both pathological blood flow (d-flow) and a stiff vascular matrix, it is logical that combining these conditions is important to the identification of translationally important pathways. Unfortunately, it is not currently known how these two environmental mechanical conditions modulate EC phenotype. Furthermore, the effect of biological sex on EC response to these PAD flow and mechanical environments has not been studied with single cell analyses.

Despite the NIH mandate to include sex as a biological variable, sex-differences have not been adequately investigated in this space (17, 18). The inclusion of biological sex as a factor in cardiovascular disease research, and in PAD research, is imperative because the onset and presentation of these diseases in humans vary with biological sex. With regards to stiffening and flow, there is an *in vitro* study that found female human umbilical vein ECs (HUVECs) were less impacted than male HUVECs when exposed to higher and lower laminar shear stress and increasing substrate stiffness since they presented no change in morphology and YAP1 nuclear translocation (19). In parallel, we have been integrating substrate stiffness (softer and stiffer reflecting stiffness of healthy and diseased arteries) with low and oscillatory wall shear stress (mimicking the d-flow environment around blockages in PAD) and using laminar wall shear stress (stable flow) to reflect non-obstructed flow. Here, we incorporate scRNA-seq methodologies to enable cell specific gene expression data, which cannot be harvested using bulk RNA sequencing. The objective of this study is to rigorously investigate the impact of stiffness and flow on sex differences in the *in vitro* response of human aortic ECs (HAECs) in the context of PAD conditions (stiff substrate and d-flow). Given the powerful independent effect of stiffness or flow on ECs, we hypothesized that scRNA-seq would reveal sex-based phenotypic differences under PAD biomechanical conditions that may contribute to sex-based differences in PAD.

## 2. Methods

### 2.1. HAECs culturing and shear stress exposure

HAECs from three age-appropiate male (ages 46, 54, and 63) and two female (ages 46 and 53) donors (Lot#: 2,895, 2,824, 1,622, 1,576, 1,851; Cell Applications, San Diego, CA) were expanded, cultured, and sheared in complete endothelial cell growth medium (Cat.# 211-500; Cell Applications, San Diego, CA). HAECs of passages 4–6 were seeded onto modified 6-well plates functionalized with collagen-coated hydrogels (Cat.# SW6-COL-25 and SW6-COL-100; Matrigen, Irvine, CA) of different stiffnesses (25 kPa and 100 kPa) that mimic the EC environment in physiologic (25 kPa) and PAD conditions (100 kPa) (20, 21). The HAECs were sheared utilizing a modified orbital shaker method (11, 22, 23). In brief, hydrogel-containing 6-well plates were modified by preventing HAEC growth in the outer (> 10.5 mm) or inner (< 10.5 mm) portion of the well by placing autoclaved vinyl obstructions prior to seeding the ECs. HAECs grown in the outer ring of the well mimicked laminar shear or stable flow (LS, timed-averaged shear stress of 7 dynes/cm^2^) while HAECs grown in the inner circle mimicked disturbed flow (OS, timed-averaged shear stress of 3 dynes/cm^2^). The cells were sheared for 96 h utilizing an orbital shaker at 100 rpm, changing media every two days. For comparison, HAECs cultured on tissue culture plastic (TCP) immediately prior to subculture conditions above were analyzed. ECs on TCP (2 GPa) and static conditions are known to mimic many of the pathways seen under s-flow conditions, including *THBS1* (9, 24).

### 2.2. Single-cell collection

Following exposure to shear stress, HAECs were recovered utilizing TrypLE (Cat.# 12563011; Gibco, Thermo Fisher Scientific, Waltham, MA) and suspended in 1X PBS (Cat.# MT21040CV) with 0.04% BSA. After ensuring the proper cell concentration was collected, the samples were delivered to the Emory Integrated Genomics Core for single-cell processing and barcoding with the 10X Genomics Chromium device. NovaSeq S4 was utilized to prepare the libraries and sequence the samples. Then, the CellRanger software was utilized to demultiplex and align the sequenced data.

### 2.3. scRNA-seq analysis and gene ontology

scRNA-seq data was processed and analyzed using the Seurat package in R (RRID:SCR_016341). To remove low-quality cells or cell multiplets, standard quality control filters were applied (11). Cells containing more than 8,000 or less than 200 unique feature counts were removed. In addition, cells containing more than 15% mitochondrial counts were removed from the analysis as dying cells often express mitochondrial contamination. Next, the data was normalized and scaled. The data pertaining to the static culture group was merged across donors, subjected to unsupervised clustering, and visualized with UMAPs, dot plots, and heatmaps. The data pertaining to all shearing conditions and the static culture group were merged for each HAEC donor and the data was visualized using violin plots and feature plots. Finally, we utilized gene ontology (GO) analysis to identify pathways enhanced in the different shear, stiffness, and biological sex groups of HAECs. GO analysis was performed by obtaining the top 200 differentially expressed genes across the different groups. Then, we utilized PANTHER to identify the GO biological process terms enhanced in each of the groups. We plotted the top 10 upregulated GO terms per condition, detailing the fold enrichment.

### 2.4. In vivo validation of EC Sex-differences: PCL surgery and qPCR

Partial carotid artery ligation (PCL) surgery was performed on 9 to 15-week-old S129 and C57BL/6 wild type (WT) mice as previously published (7, 25). The left carotid artery (LCA) was subjected to d-flow by PCL while the right carotid artery (RCA) served as *in situ* control. D-flow has been shown to induce arterial stiffening similar to that seen in aged 80-week-old animal (9). 24 h following PCL, EC-enriched RNA was collected from the carotid arteries as previously described (7, 25). 2–3 mouse arteries were pooled for each sample (n) and analyzed. RNA was isolated from the cell lysate and One-Step SYBR green (Cat.# 1725150; Bio-Rad, Hercules, CA) was used for gene analysis utilizing quantitative real-time polymerase chain reaction (qPCR, RT-PCR). The primers used were *CCN2* and *THBS1* with *18S* as the housekeeping gene (Integrated DNA Technologies, USA). The gene analysis for the *in vivo* validation was presented as the fold change normalized against *18S*.

### 2.5. Statistical analysis

The validation data collected from quantitative PCR was analyzed utilizing GraphPad Prism. Each data point represents 2–3 LCAs or RCAs from mouse following 24 h of PCL. The fold change gene expression of the two WT strains utilized here (S129 and C57BL/6) were pooled for plotting. A two-sample Wilcoxon-Mann–Whitney test was done to compare between biological sex. Significance was set as a *p*-value of <0.05.

## 3. Results

### 3.1. Sex-based differences identified in HAECs in static culture

**Figure 1:**
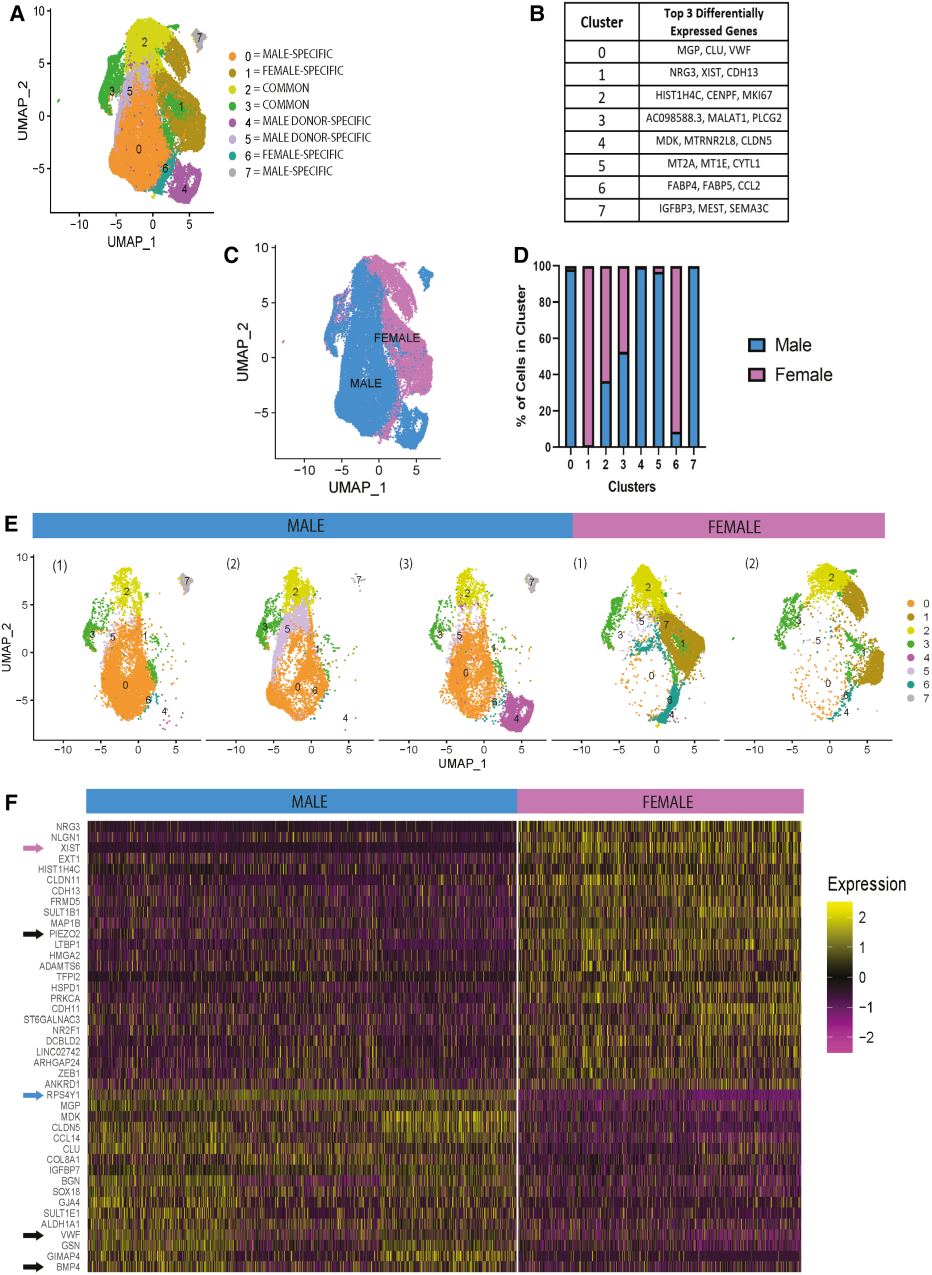
In static conditions, gene expression of HAECs is different across biological sex. (**A–F**) scRNA-seq data for HAECs cultured in static conditions. (**A**) UMAP presenting the 8 distinct clusters identified. (**B**) Table showing top three differentially expressed genes per cluster. (**C**) UMAP identifying female and male HAECs. (**D**) Histogram presenting the percentage of HAECS that are male or female per cluster. (**E**) UMAPs separated by donor, *n* = 3 male, *n* = 2 female. (**F**) Following differential gene expression analysis, a pseudo-bulk heat map was generated showing the top 30 differentially expressed genes for the male and female HAECs group.

To identify sex-specific differences in static conditions, HAECs on TCP and under static culture were pooled across donors. This condition mimics how ECs are typically expanded. After pooling all donors, eight distinct clusters were identified following unsupervised clustering and are shown using a UMAP plot (Figure 1A). The clusters were defined to be donor-specific, sex-specific, or common to all donors. Clusters that were donor-specific are those only present in specific HAEC donors. For example, clusters 4 and 5 are only found in male donors 3 and 2, respectively (Figure 1E). Figure 1B shows the top three differentially expressed genes per cluster, indicating HAEC heterogeneity in static culture. As shown in Figure 1C, many clusters were, in part, organized by the donor's biological sex. The histogram in Figure 1D confirms that some clusters, as presented in Figure 1A, are sex specific. Then, we evaluated donor heterogeneity in static culture by viewing UMAPs separated by donor (Figure 1E) and found no considerable differences within donors of the same sex, except those already summarized in Figure 1A. These UMAPs show clear transcriptional sex-specific differences in HAECs in static culture. Next, we performed a differential gene (pseudo-bulk RNA) expression analysis to identify the sex-specific differences in our scRNA-seq data of HAECs under static culture. The top 30 differentially expressed genes ranked by normalized expression in male and female HAECs are presented in the heatmap plot (Figure 1F). As expected, sex-linked genes like *XIST* and *RPS4Y1* rank among the highest in female and male, respectively. However, other non-sex-linked genes were identified. Specifically, in male HAECs, genes like *VWF* and *BMP4* are differentially expressed, while in female HAECs, *PIEZO2* was increased.

### 3.2. Sex-specific differential response of HAECs exposed to shear stress and substrate stiffness

**Figure 2:**
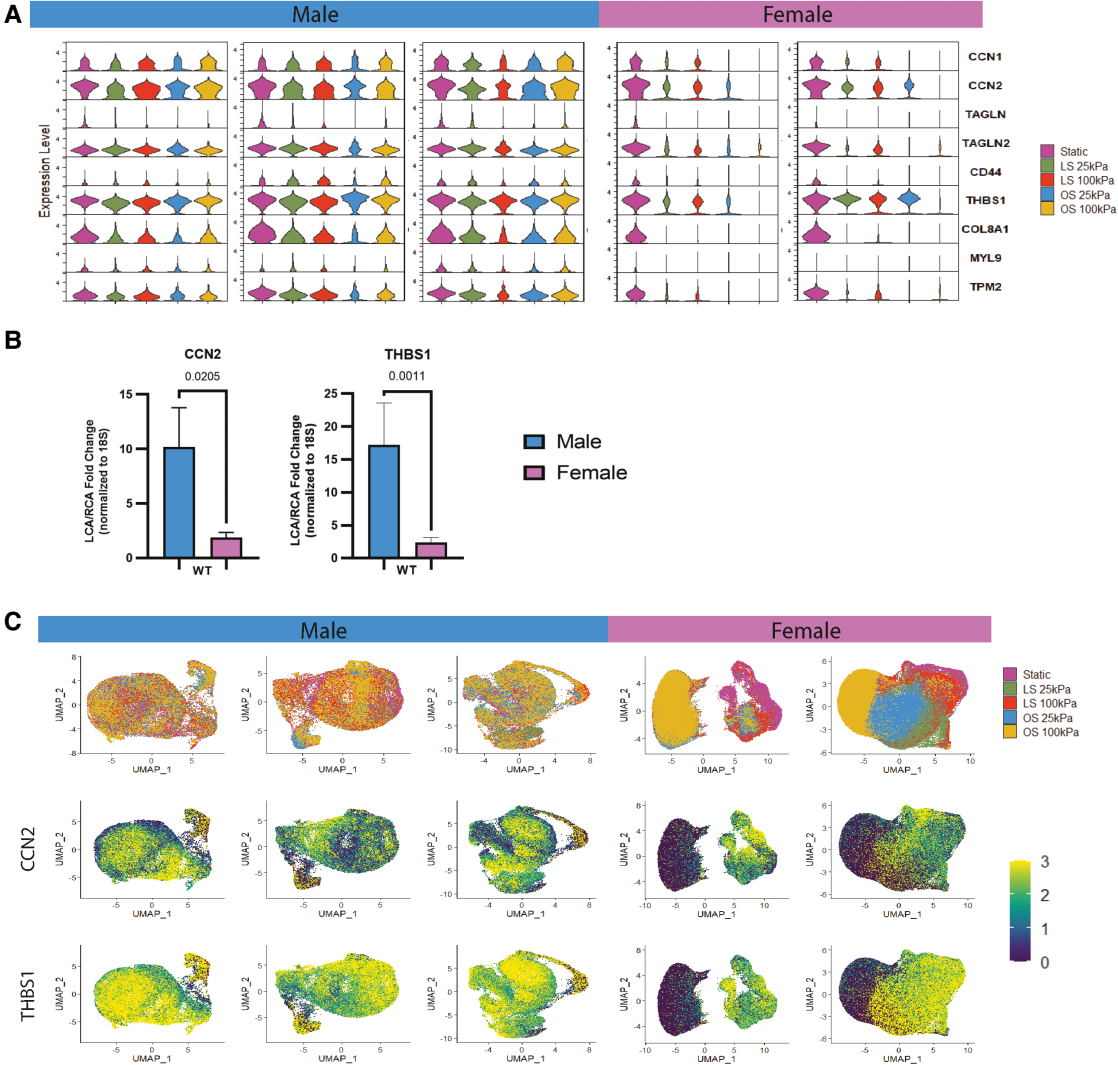
Male and female HAECs respond differently to shear and substrate stiffness. (**A**) Violin plots from scRNA-seq data showing the different gene expressions of female and male HAECs under laminar shear (LS), oscillatory shear (OS) or static culture and seeded on soft (25 kPa) and stiff (100 kPa) substrates. (**B**) *In vivo* validation of male and female scRNA-seq analysis showing fold change differences in *CCN2* and *THBS1* in EC-enriched RNA of the d-flow left carotid artery (LCA) normalized to the s-flow right carotid artery (RCA) of mice after 24 h. D-flow in LCA was imposed by partial carotid ligation. The data is presented as the mean ± SEM (*n* = 3-5). (**C**) The top row shows donor-specific UMAP plots merging all shear and substrate stiffness conditions. The bottom two rows are feature plots from scRNA-seq in each donor detailing *CCN2* and *THBS1* expression level merging all shear and substrate stiffness conditions.

Motivated by the sex-specific differences shown in the static culture group, we next investigated the existence of sex-specific differences when HAECs were exposed to different magnitudes of shear stress and substrate stiffness. The relative gene expression across all shear stress and substrate conditions per donor was plotted in violin plots (Figure 2A). Of interest, we can note that the gene expression level of HAECs in response to mechanical stimuli was stronger in males than in females. This is particularly notable when evaluating profibrotic genes like *CCN1*, *CCN2*, *COL8A1* and d-flow associated genes like *THBS1* that present higher expression in male HAECs after mechanical stimuli.

### 3.3. In vivo validation

We validated two important fibro-inflammatory markers from our *in vitro* scRNA-seq findings with two commonly used WT murine strains (S129 and C57BL/6). Here, EC-enriched RNA was collected after 24 h of d-flow, induced by PCL in the LCA; the contralateral RCA serving as an *in situ* control (7, 25). *CCN2* and *THBS1* expression levels were quantified by qPCR (Figure 2B). Fold change gene expressions of both WT strains were merged. *CCN2* and *THBS1* expression was significantly higher in males than females. These trends were consistent with our scRNA-seq data (Figure 2C) which show higher gene expression levels in male HAECs when compared to female HAECs for *THBS1* and *CCN2*. The UMAP and feature plots shown in Figure 2C allow for the evaluation of which HAECs across the different shear and substrate conditions are expressing more *CCN2* and *THBS1*. Moreover, the degree of expression of these genes is consistent across scRNA-seq *in vitro* and bulk *in vivo* data, with *THBS1* being abundantly expressed followed by *CCN2*.

### 3.4. GO analysis

**Figure 3:**
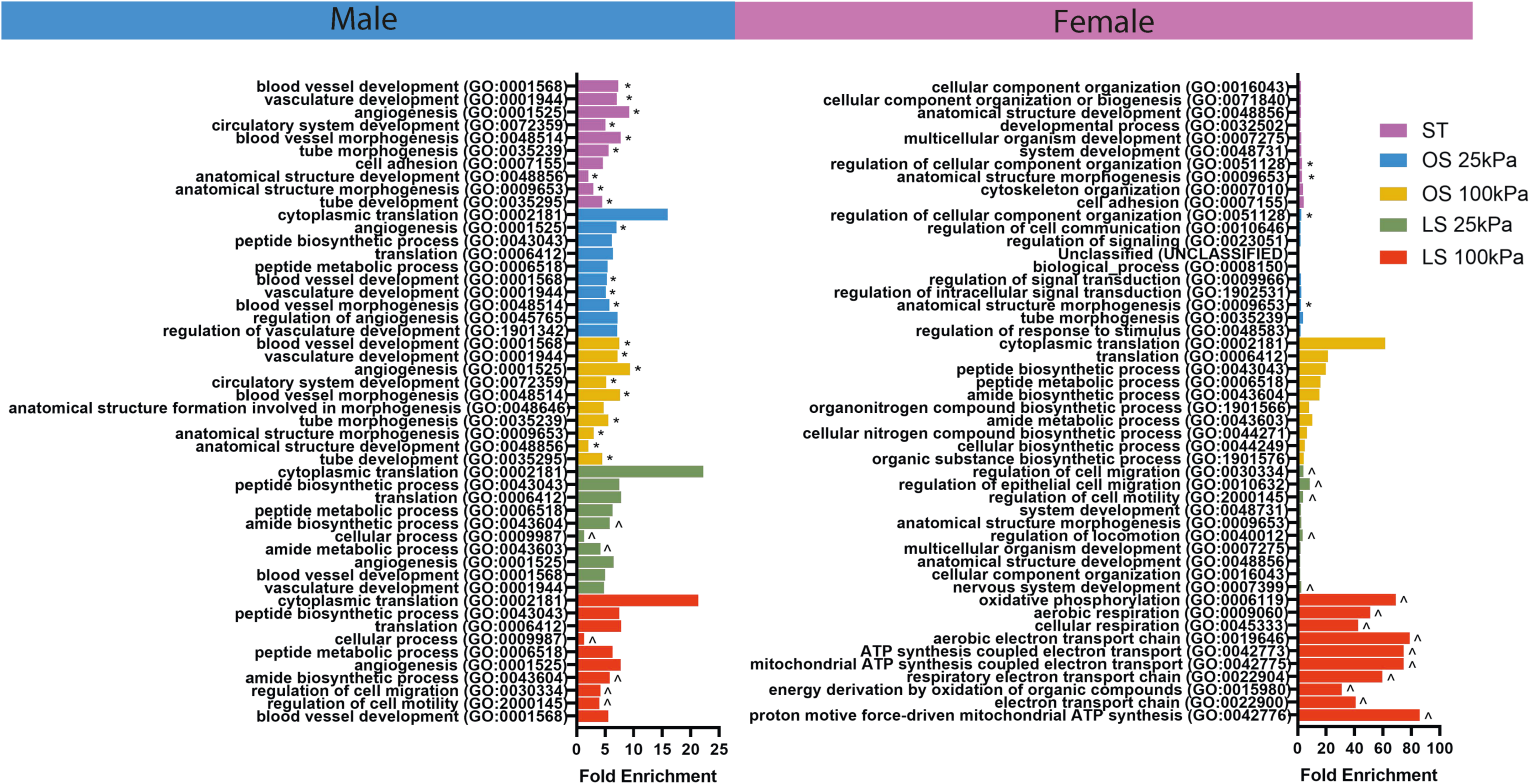
Male HAECs share more GO terms across static and OS conditions while female HAECs show more unique GO terms in LS groups. GO analysis results showing the top 10 most significant GO biological process terms across stiffness, shear, and biological sex conditions. GO was performed by obtaining the top 200 differentially expressed genes across stiffness, shear, and biological sex conditions. Asterisks (*) indicate the GO terms that are shared between the static culture, OS 25 kPa, and OS 100 kPa groups within biological sex. The circumflex symbols (^) denote the GO terms that were unique to the LS 25 kPa and LS 100 kPa groups within biological sex.

To test relevant signaling pathways, we evaluated enhanced GO terms in each condition across biological sex (Figure 3). We found that male HAECs exposed to a mechanical environment mimicking PAD exhibited many of the EndMT pathways associated with vasculature development while female HAECs showed processes related to cellular signaling and metabolism. Moreover, when we evaluated the GO terms shared between the static, OS 25 kPa, and OS 100 kPa groups for each biological sex (delineated with * in Figure 3), we found a large overlap of GO terms in males and less so in female HAECs. We also investigated the GO terms unique to the LS 25 kPa and LS 100 kPa groups for each biological sex (marked with ^ in Figure 3) and discovered more unique LS GO terms in females compared to males.

## 4. Discussion

This study leveraged scRNA-seq analysis to investigate phenotypic differences by cell-specific gene expression between male and female HAECs under clinically relevant mechanical environments (softer/stiffer substrates; laminar/oscillatory shear stress). After finding distinct differences between male and female HAECs on TCP under static culture conditions, we considered the effect of mechanical stimuli on sex-specific differences. We found that male HAECs presented higher expression of genes related to fibrosis and d-flow. Lastly, we validated our scRNA-seq results with *in vivo* results from a murine PCL model of d-flow. The EC-enriched RNA taken from these arteries showed gene expressions consistent with those found in the *in vitro* model that was analyzed with scRNA-seq.

This concise study opens the door to several avenues of investigation. While our findings clearly showed male-female differences in gene expression for HAECs exposed to mechanical stimuli, gene expression differences across LS and OS groups are not as clear. Particularly, common EC d-flow markers such as *CCN2*, *TAGLN*, and *THBS1* (9, 11) that at this time point, did not show marked upregulation by OS. Since these d-flow genes have not been previously studied in HAECs cultured on substrates of different stiffnesses, future work will address temporal changes that may be important to this process. This is easily incorporated into this model system that innovatively incorporates stiffness/flow mimicking PAD conditions *in vitro*, and *in vivo* models can match such time points for translational testing. Understanding how the various mechanical stimuli differentially affect molecular pathways within ECs is of interest to our group, particularly how a PAD environment that includes a stiff ECM and d-flow leads to atherosclerosis onset and progression. In this regard, our *in vitro* scRNA-seq and *in vivo* data shows a clear sex-based difference in *THBS1* and *CCN2*, which are currently being investigated as part of fibroinflammatory pathways in PAD (9). In addition, further investigation of our pathway enrichment analysis is ongoing, and we expect to be able to discover gene groups and molecular pathways could play important roles in PAD.

This study included 5 HAEC donors (3 male and 2 female), which is similar in donor number to many publications (26–28). All donors were in the age range for PAD (46–63 years). Nonetheless, to investigate if genetic factors in donors significantly affect our conclusions more HAEC donors would be needed. To date, we are not sure of the importance of donor age on the findings presented here, and commercially available donors are quite limited. This is particularly so for aged donors. As such, we plan to use our access to patients and patient tissue in future studies that utilize scRNA-seq on PAD patient arteries under d-flow and s-flow conditions. Arteries will be further segregated by stiffness using mechanical testing as published (9, 29–31). This will permit us to validate key findings and discover new pathways that may lead to translational therapies to better treat both male and female PAD patients.

Others have previously utilized scRNA-seq analysis tools to examine the effects of the mechanical environment of ECs on the transcriptome at a single-cell resolution as it relates to atherosclerosis, but biological sex was not taken into consideration (11, 16). A recent study by Zamani et al. used scRNA-seq analysis to show that ECs cultured on TCP presented high heterogeneity and higher mesenchymal transcriptional features suggesting EndMT progression (16). Similarly, our group of HAECs exposed to no flow and cultured on tissue culture plastic presented high heterogeneity (Figure 1). Moreover, our static culture group showed high gene expression of smooth muscle cell and mesenchymal cell markers like *TAGLN*, *TAGLN2*, *MYL9*, and *TPM2* in male and female HAECs (Figure 2A). This might be due to the high stiffness of the tissue culture plastic which, as proposed by Zamani et al., could lead to EndMT progression. It is well known that static culture on TCP has a genetic signature very similar to that of disturbed flow and in contrast to that of stable flow (9, 32). The idea of arterial stiffness being involved in atherosclerosis progression has been studied previously and with our experimental design we can begin investigating how biological sex could play a role in this (16, 33, 34). The work we have done here also takes into consideration both the pathological mechanical and hemodynamic microenvironment provided by a stiff ECM and disturbed flow conditions. This ensured that the pathological conditions presented in atherosclerotic lesions *in vivo* are more accurately represented in the *in vitro* model utilized. Furthermore, our study centers on the effect of biological sex on the HAEC response to shear stress and substrate stiffness, which appears to be a key component of EC response to flow and stiffness that has been largely overlooked in prior publications.

Biological sex is underreported in *in vitro* and *in vivo* research studies in the cardiovascular field (17, 18). In the case of PAD, the inclusion of biological sex as a study variable might help explain the delayed onset of PAD in females. Recent work by James and Allen shows the imperative need of including biological sex as an experimental variable in *in vitro* studies. After applying controlled shear stress (15 dynes/cm^2^ or 5 dynes/cm^2^) to HUVECs seeded on substrates of different stiffnesses (10 kPa or 100 kPa), they discovered that female HUVECs remained mostly invariant to the different combinations of mechanical stimuli suggesting that females might be more resistant to changes in the mechanical environment of ECs (19). Here, we exposed female HAECs to a complex mechanical environment mimicking PAD and found an attenuated response in comparison to males. This is consistent with the delayed onset of PAD in females. In contrast, transforming growth factor beta (*TGF-β*) has been proposed to be important to atherosclerotic remodeling, but the data presented was all from male animals (35). Since *THBS1* can activate *TGF-β*, this mechanism may be relatively more important in males compared to females. Biological sex-based differences are present in cardiovascular diseases such as pulmonary arterial hypertension, aortic valve stenosis, PAD, and more (6, 26, 36). Studies investigating sexual dimorphisms in cardiovascular disease have queried genes encoded in sex chromosomes since recent findings suggested that non-hormonal differences might affect the differential disease presentation observed (37, 38). For gene dosage balance, female cells undergo X chromosome inactivation (XCI). Failure to do so could lead to the unbalanced transcription of X-chromosome genes (27). The effect this could have on cardiovascular disease is being investigated, but no studies have examined XCI in PAD. This is a potential and exciting future direction for our work. These intrinsic biological differences warrant the specification of sex in studies utilizing cells. Furthermore, if sex-specific differences are heightened by the experimental conditions, in our case, mechanical stimulation, future research must mandate better inclusion of biological sex as a variable in experimental design.

This work highlights significant sex-based EC phenotypic differences induced by various clinically relevant culture conditions that may have profound impact on our understanding of sex-based clinical differences. In particular, this study found that male HAECs on TCP and cultured under static conditions and HAECs exposed to OS conditions had relatively similar gene expression in markers of EndMT and mesenchymal cells. In contrast, female HAECs showed higher EndMT and mesenchymal cell markers in HAECs cultured on static conditions when compared to other shear and substrate stiffness conditions. The *in vitro* model utilized in this work also enables rigorous delineation of sex-based EC differences in a setting that can exclude hormonal interactions of *in vivo* studies as well as comparisons between humans and mice that have distinct hormonal patterns across all ages. Still, this *in vitro* platform could be used to incorporate exogenous hormone levels to test *in vivo* conditions. Further, our *in vivo* validation supports acute sex-based EC expression differences under PAD flow conditions.

In conclusion, sex-based differences are seen in HAECs under PAD conditions. These EC phenotypes may contribute to sex-based clinical differences in PAD. The use of scRNA-seq is a powerful technique that will allow better understanding of ECs under complex biomechanical conditions. Future work may further aid in the discovery of EC heterogeneity, genetic differences of PAD patients, as well as sex-based donor differences under static culture and under clinically relevant conditions.

## Data Availability

The data presented in this study are deposited in the NCBI BioProject repository with accession number: PRJNA1008616. The data is available at: https://www.ncbi.nlm.nih.gov/bioproject/PRJNA100861.
